# Structures of Dimer-of-Dimers Type Defect Cubane Tetranuclear Copper(II) Complexes with Novel Dinucleating Ligands

**DOI:** 10.3390/molecules27020576

**Published:** 2022-01-17

**Authors:** Ryusei Hoshikawa, Ryoji Mitsuhashi, Eiji Asato, Jianqiang Liu, Hiroshi Sakiyama

**Affiliations:** 1 Department of Science, Faculty of Science, Yamagata University, 1-4-12 Kojirakawa, Yamagata 990-8560, Yamagata, Japan; hosiryu@yahoo.com; 2 Institute of Liberal Arts and Science, Kanazawa University, Kakuma, Kanazawa 920-1192, Ishikawa, Japan; mitsuhashi@staff.kanazawa-u.ac.jp; 3 Department of Chemistry, Biology and Marine Science, Faculty of Science, University of the Ryukyus, Nishihara 903-0213, Nakagami-gun, Okinawa, Japan; asato@sci.u-ryukyu.ac.jp; 4 Guangdong Provincial Key Laboratory of Research and Development of Natural Drugs, School of Pharmacy, Guangdong Medical University, Guangdong Medical University Key Laboratory of Research and Development of New Medical Materials, Dongguan 523808, China; jianqiangliu2010@126.com

**Keywords:** tetranuclear copper(II) complex, dinucleating ligand, dimer-of-dimers type, crystal structure, magnetic properties, density functional theory (DFT)

## Abstract

Only a limited number of multinucleating ligands can stably maintain multinuclear metal structures in aqueous solutions. In this study, a water-soluble dinucleating ligand, 2,6-bis{[*N*-(carboxylatomethyl)-*N*-methyl-amino]methyl}-4-methylphenolate ((*sym*-cmp)^3−^), was prepared and its copper(II) complexes were structurally characterized. Using the single-crystal X-ray diffraction method, their dimer-of-dimers type defect cubane tetranuclear copper(II) structures were characterized for [Cu_4_(*sym*-cmp)_2_Cl_2_(H_2_O)_2_] and [Cu_4_(*sym*-cmp)_2_(CH_3_O)_2_(CH_3_OH)_2_]. In the complexes, each copper(II) ion has a five-coordinate square-pyramidal coordination geometry. The coordination bond character was confirmed by the density functional theory (DFT) calculation on the basis of the crystal structure, whereby we found the bonding and anti-bonding molecular orbitals. From the cryomagnetic measurement and the magnetic analysis, overall antiferromagnetic interaction was observed, and this magnetic behavior is also explained by the DFT result. Judging from the molar conductance and the electronic spectra, the bridging chlorido ligand dissociates in water, but the dinuclear copper(II) structure was found to be maintained in an aqueous solution. In conclusion, the tetranuclear copper(II) structures were crystallographically characterized, and the dinuclear copper(II) structures were found to be stabilized even in an aqueous solution.

## 1. Introduction

Copper is an essential trace element [1,2], and we humans cannot live without it. In fact, a 70 kg adult human body contains ~0.11 g of copper [1]. Humans need oxygen for cellular respiration to extract energy from food, and for cellular respiration, cytochrome *c* oxidase requires iron and copper to bind and activate oxygen [1,2,3]. In addition, toxic superoxide is produced daily together with cellular respiration, and superoxide dismutase (SOD) requires copper and zinc to decompose superoxide [1,2,3]. These are just a few examples of copper enzymes, and various copper proteins and copper enzymes play important roles in life. Some of the copper proteins have two or more copper ions at the active site and have functions that cannot be achieved by one copper ion. For the purpose of artificially realizing the function of such multi-copper proteins, many multinucleating ligands have been developed to stabilize the multinuclear metal complex structures.

**Figure 1:**
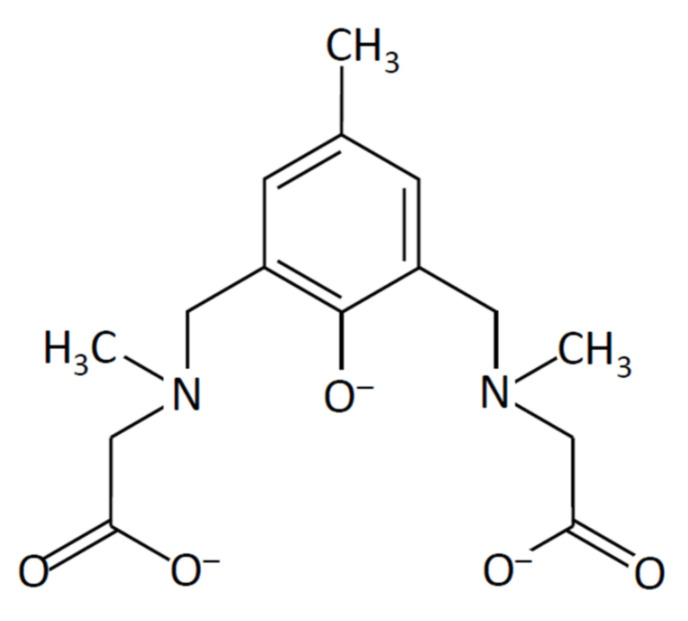
Chemical structure of (*sym*-cmp)^3−^.

2,6-Bis[bis(2-pyridylmethyl)aminomethyl]-4-methylphenol (H(bpmp)) is one of the most well-known acyclic dinucleating ligands, providing two N_3_O coordination sites [4]. *N*,*N*’-(2-Hydroxy-5-methyl-1,3-xylylene)bis[*N*-(carboxymethyl)glycine] (H_5_(5-Me-hxta)) is another well-known acyclic dinucleating ligand, possessing two NO_3_ coordination sites [5,6]. Both ligands, (bpmp)^−^ and (5-Me-hxta)^5−^, are end-off type acyclic dinucleating ligands with a phenolato moiety as a bridging group and are suitable for incorporating various dinuclear metal cores. In addition, metal complexes with these ligands and their derivatives [7] are stable in aqueous solutions, while Schiff base ligands are often hydrolyzed in aqueous solutions. When (bpmp)^−^ or (5-Me-hxta)^5−^ incorporates two octahedral metal ions, two coordination sites will be available for substrate incorporation in catalytic reactions. In this study, for the purpose of increasing the number of available coordination sites, a novel dinucleating ligand, 2,6-bis{[*N*-(carboxylatomethyl)-*N*-methyl-amino]methyl}-4-methylphenolate ((*sym*-cmp)^3−^) was synthesized (Figure 1). The ligand (*sym*-cmp)^3−^ has a bridging phenolato moiety and two NO_2_ coordination sites and is expected to incorporate two metal ions. This paper reports the crystal structures of dimer-of-dimers type tetranuclear copper(II) complexes with (*sym*-cmp)^3−^.

## 2. Results and Discussion

### 2.1. Preparation

#### 2.1.1. Preparation of a Dinucleating Ligand

A dinucleating ligand, 2,6-bis{[*N*-(carboxylatomethyl)-*N*-methyl-amino]methyl}-4-methylphenolate ((*sym*-cmp)^3−^) was synthesized via a Mannich reaction from *p*-cresol and sarcosine. The ligand was obtained as a sodium salt and recrystallized from ethanol. The ligand was characterized by IR, elemental analysis, ^1^H and ^13^C NMR, and electrospray ionization (ESI) mass spectrometry. In ^1^H NMR, five singlet signals characteristic for (*sym*-cmp)^3−^ were obtained (Figure S1), and in ^13^C NMR, nine characteristic signals were obtained (Figure S2). In ESI-mass spectra, the main peak at *m*/*z* = 309 was assigned to [H_2_(*sym*-cmp)]^−^ (Figure S3), and its elemental composition (C_15_H_21_N_2_O_5_) was confirmed by the isotope pattern (Figure S4). Judging from the small molar conductance value (19 S·cm^2^·mol^−1^) in water, the sodium ions are considered to be tightly incorporated in the ligand.

#### 2.1.2. Preparation of Copper(II) Complexes

With the dinucleating ligand (*sym*-cmp)^3−^, tetranuclear copper(II) complexes were prepared as dimers of dinuclear copper(II) units. Using the copper(II) chloride, a chlorido complex, [Cu_4_(*sym*-cmp)_2_Cl_2_(H_2_O)_2_]·2H_2_O (**1**), was obtained, while a methoxido derivative, [Cu_4_(*sym*-cmp)_2_(CH_3_O)_2_(CH_3_OH)_2_]·2C_3_H_7_OH·2CH_3_OH (**2**), was obtained by using the copper(II) nitrate. Purification of **2** was very difficult, and the crude product often contains nitrate ions. So, complex **2** was characterized only by the single-crystal X-ray diffraction method. Structural details will be described in the following crystallographic section (Section 2.2) and the theoretical calculation section (Section 2.4).

### 2.2. Crystal Structures of Copper(II) Complexes

#### 2.2.1. Crystal Structures of [Cu_4_(*sym*-cmp)_2_Cl_2_(H_2_O)_2_]·2.4CH_3_OH·1.8H_2_O (**1′**)

**Figure 2:**
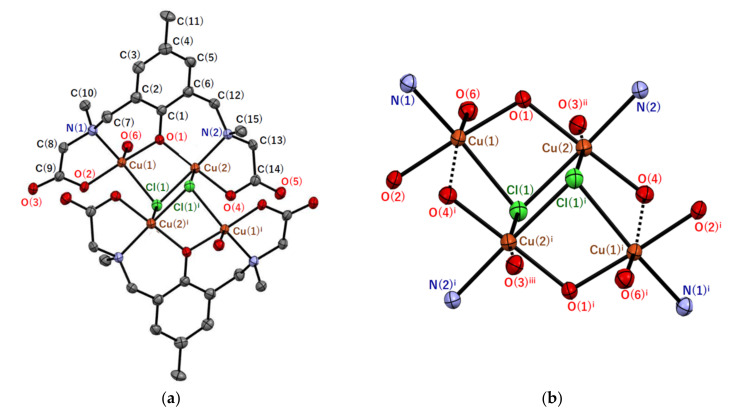
Molecular structures of (**a**) [Cu_4_(*sym*-cmp)_2_Cl_2_(H_2_O)_2_] and (**b**) Cu_4_Cl_2_N_4_O_10_ core in **1**′ with atom labeling. Hydrogen atoms are omitted for clarity. Thermal ellipsoids are drawn at the 50% probability level. Symmetry code: ^i^ (−*x* + 1/2, −*y* + 1/2, −*z* + 1), ^ii^ (−*x* + 1/2, *y* + 1/2, −*z* + 3/2), ^iii^ (*x*, −*y*, *z* − 1/2).

Single crystals of **1′** were obtained by recrystallization of **1** from methanol. (Note that **1** is a dried sample, while **1′** is a sample in a crystalline state where drying was prevented.) Although **1** was considered to contain two water molecules as solvent of crystallization per tetranuclear copper(II) unit from the elemental analysis, solvents of crystallization of **1′** were empirically determined as 2.4 methanol and 1.8 water molecules. The crystal structure of the tetranuclear copper(II) complex [Cu_4_(*sym*-cmp)_2_Cl_2_(H_2_O)_2_] and its tetranuclear bridging structure are shown in Figure 2, and selected atomic distances and angles are summarized in Table 1 and Table 2.

The tetranuclear copper(II) complex [Cu_4_(*sym*-cmp)_2_Cl_2_(H_2_O)_2_] is centrosymmetric (Figure 2a) and considered as a dimer-of-dimers type tetranuclear copper(II) complex, possessing the defect cubane tetranuclear copper(II) core (Figure 2b). Each dinucleating ligand, (*sym*-cmp)^3−^, incorporates two copper(II) ions bridged by one phenolic oxygen of the dinucleating ligand and by one chlorido ligand. If we consider only the typical coordination bonds, each copper(II) ion has five-coordinate square-pyramidal coordination geometry, and Cu(1) and Cu(2) ions are surrounded by NO_3_Cl and NO_2_Cl_2_ donor sets, respectively. The distortion parameter *τ* defined as *τ* = (*θ* − *φ*)/60 × 100% is calculated as 20.9% for Cu(1) and 6.1% for Cu(2), where *θ*° and *φ*° are the largest and the second largest bond angles around each copper atom, respectively. The parameter *τ* is 100% if the coordination geometry is purely trigonal–bipyramidal, while *τ* is 0% if the geometry is purely square–pyramidal. Therefore, both coordination geometries are considered to be square–pyramidal. Each of the apical bond distances (Cu(1)–O(6) = 2.222(3) Å and Cu(2)–Cl(1)^i^ = 2.8011(13) Å) is longer than the other basal Cu–O (1.925(3)–1.943(3) Å) and Cu–Cl (2.3124(13)–2.3630(12) Å) bond lengths, respectively. This can be explained by the Jahn–Teller effect [8], typical for the copper(II) complexes with *d*^9^ electronic configuration.

The apical Cu–Cl distance (Cu(2)–Cl(1)^i^ = 2.8011(13) Å) may seem to be slightly too long for the coordination bond; however, the covalent bond character was confirmed by the density functional theory (DFT) calculation (Section 2.4). Therefore, the chlorido ligand is definitely bridging three copper(II) ions, forming the defect cubane tetranuclear copper(II) core structure. On the other hand, two more weak coordination bonds were found by DFT calculations (dashed bonds in Figure 2b). One is the Cu(1)–O(4)^i^ bond (3.209(3) Å), and the other is the Cu(2)–O(3)^ii^ bond (2.804(4) Å) between Cu(2) and an oxygen atom in a neighboring tetranuclear copper(II) complex. Here, the weak coordination bond refers to a bond with less covalency than the typical coordination bond, where the overlap of atomic orbitals involved in the bond is smaller. When the weak coordination bonds are also taken into account, the coordination geometries around the two copper(II) ions are both octahedral.

Tetranuclear copper(II) complexes with similar tetranuclear copper(II) cores are reported [9,10,11], and their magnetic properties were analyzed. In two of them, all three adjacent copper(II) pairs are doubly bridged [9,10], while in the rest of them, two of the adjacent copper(II) pairs are doubly-bridged ones, but one pair is singly-bridged [11]. In a precise sense, this type of core structure is often called the stepped cubane, but in the case of **1′**, the core structure can be included in the defect cubane.

#### 2.2.2. Crystal Structures of [Cu_4_(*sym*-cmp)_2_(CH_3_O)_2_(CH_3_OH)_2_]·2C_3_H_7_OH·2CH_3_OH (**2**)

**Figure 3:**
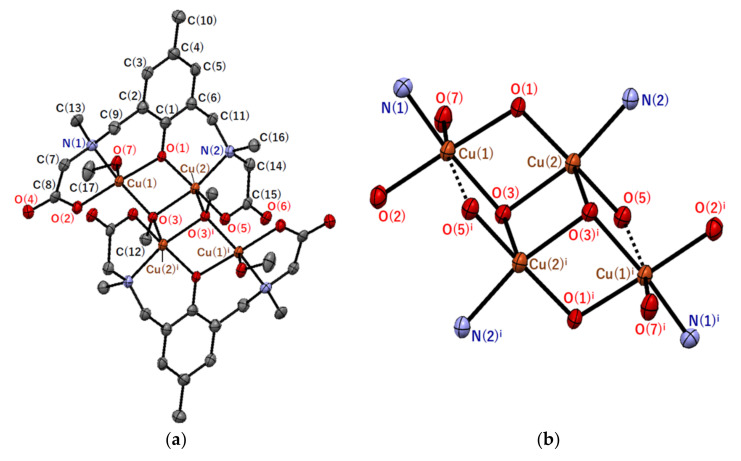
Molecular structures of (**a**) [Cu_4_(*sym*-cmp)_2_(CH_3_O)_2_(CH_3_OH)_2_] and (**b**) Cu_4_N_4_O_10_ core in **2** with atom labeling. Hydrogen atoms are omitted for clarity. Thermal ellipsoids are drawn at the 50% probability level. Symmetry code: ^i^ (−*x* + 1, −*y* + 1, −*z* + 1).

Single crystals of **2** were obtained by slow diffusion of 2-propanol to a methanolic solution of the crude product. The crystal structure of the tetranuclear copper(II) complex [Cu_4_(*sym*-cmp)_2_(CH_3_O)_2_(CH_3_OH)_2_] and its tetranuclear bridging structure are shown in Figure 3, and selected atomic distances and angles are summarized in Table 3 and Table 4.

The basic skeletal structure of the tetranuclear copper(II) complex [Cu_4_(*sym*-cmp)_2_(CH_3_O)_2_(CH_3_OH)_2_] in **2** is very similar to the complex structure in **1′**. That is, the bridging chlorido and water ligands in **1′** are replaced with methoxido and methanol ligands, respectively. The [Cu_4_(*sym*-cmp)_2_(CH_3_O)_2_(CH_3_OH)_2_] complex is centrosymmetric (Figure 3a) and considered a dimer-of-dimers type tetranuclear copper(II) complex, possessing the defect cubane tetranuclear copper(II) core (Figure 3b). A pair of copper(II) ions incorporated into one dinucleating ligand are bridged by one phenolic oxygen of the dinucleating ligand and by one methoxido ligand. Both types of copper(II) ions have five-coordinate square-pyramidal coordination geometries with NO_4_ donor atoms, if we consider only the typical coordination bonds. The distortion parameter *τ* was 16.5% for Cu(1) and 11.0% for Cu(2). The apical bond distances (Cu(1)–O(7) = 2.331(3) Å and Cu(2)–O(3)^i^ = 2.305(3) Å) are longer than the other basal Cu–O distances (1.930(3)–1.999(3) Å). The longer apical distances are consistent with the d^9^ electronic configuration of the copper(II) centers discussed in Section 2.2.1. When weak coordination bonds were also taken into account, another bond, Cu(1)–O(5)^i^ (2.856(3) Å), was found in the DFT calculation, and the coordination geometry around Cu(1) became octahedral. In contrast, no covalent nature was observed between Cu(2) and adjacent O(6)^ii^ in another complex.

### 2.3. Magnetic Properties

**Figure 4:**
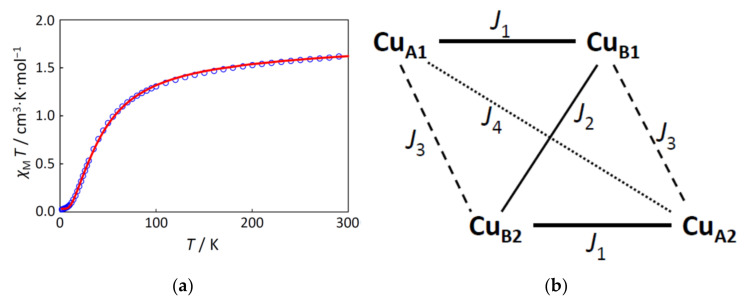
(**a**) The *χ*_M_*T versus T* plot for **1**. The observed data (○) and the theoretical curve (—) with the best-fitting parameter set (*J*_1_, *J*_2_, *J*_3_, *J*_4_, *g*, TIP, *ρ*) = (−47.9 cm^−1^, −38.5 cm^−1^, 15.3 cm^−1^, 0 cm^−1^ (fixed), 2.10, 60 × 10^−6^ cm^3^·mol^−1^ (fixed), 0.0196); (**b**) Interactions in the centrosymmetric tetranuclear copper(II) core.

The cryomagnetic behavior for complex **1** was measured for the purpose of confirming the electronic configuration of the ground state and revealing the exchange interactions between the copper(II) ions. The *χ*_M_*T* versus *T* plot is shown in Figure 4a. The observed *χ*_M_*T* product at 300 K was 1.63 cm^3^·K·mol^−1^, which was close to the spin-only value for the four independent *S* = 1/2 magnetic centers (1.50 cm^3^·K·mol^−1^). The *χ*_M_*T* value decreased on cooling to 1.9 K (0.026 cm^3^·K·mol^−1^), suggesting a strong antiferromagnetic interaction between copper(II) ions. For the magnetic analysis, the method of Hatfield and Inman [12] was used to obtain the magnetic susceptibility equation. In this study, the Hamiltonian **H** = − *J*_1_ (**S**_A1_·**S**_B1_ + **S**_A2_·**S**_B2_) − *J*_2_ **S**_B1_·**S**_B2_ − *J*_3_ (**S**_A1_·**S**_B2_ + **S**_A2_·**S**_B1_) − *J*_4_ **S**_A1_·**S**_A2_ was used (see Figure 4b). The magnetic susceptibility equations (Equations (1)–(11)) used in this study are as follows:

(1) $$\begin{array}{c} \chi_{M}=\frac{Ng^{2}\beta^{2}}{k\;T}\frac{10\;\exp\left(-\frac{A}{\;k\;T}\right)+2\;\exp\left(-\frac{B}{k\;T}\right)+2\;\exp\left(-\frac{C}{k\;T}\right)+2\;\exp\left(-\frac{D}{k\;T}\right)}{5\;\exp\left(-\frac{A}{k\;T}\right)+3\;\exp\left(-\frac{B}{k\;T}\right)+3\;\exp\left(-\frac{C}{k\;T}\right)+3\;\exp\left(-\frac{D}{k\;T}\right)+\exp\left(-\frac{E}{k\;T}\right)+\exp\left(-\frac{F}{k\;T}\right)}(1-\rho )\\ +\;\frac{Ng^{2}\beta^{2}}{k\;T}\rho +4\;\mathrm{TIP}, \end{array}$$

(2) $$A=-\frac{K}{2}-Q,$$

(3) $$B=-\frac{K}{2}+Q,$$

(4) $$C=\frac{K}{2}-\sqrt{L^{2}+P^{2}},$$

(5) $$D=\frac{K}{2}+\sqrt{L^{2}+P^{2}},$$

(6) $$E=\frac{K}{2}+Q-\sqrt{K^{2}+3L^{2}-2KQ+Q^{2}},$$

(7) $$F=\;\frac{K}{2}+Q+\sqrt{K^{2}+3L^{2}-2KQ+Q^{2}},$$

(8) $$K=\frac{J_{2}+J_{4}}{2},$$

(9) $$L=\frac{J_{1}-J_{3}}{2},$$

(10) $$P=\frac{J_{2}-J_{4}}{2},$$

(11) $$Q=\frac{J_{1}+J_{3}}{2},$$

where TIP and *ρ* are the temperature-independent paramagnetism per copper and the paramagnetic impurity with *S* = 1/2, respectively.

As the result, the best fitting parameter set was found to be (*J*_1_, *J*_2_, *J*_3_, *J*_4_, *g*, TIP, *ρ*) = (−47.9 cm^−1^, −38.5 cm^−1^, 15.3 cm^−1^, 0 cm^−1^ (fixed), 2.10, 60 × 10^−6^ cm^3^·mol^−1^ (fixed), 0.0196) with a good discrepancy factor (*Rχ* = 7.2 × 10^−5^). Overall, the magnetic interaction is antiferromagnetic, but the strongest antiferromagnetic interaction is considered to occur between the copper(II) ions in the same dinucleating ligand, expressed as *J*_1_ (=−47.9 cm^−1^), bridged by one phenolato oxygen and one chlorido chlorine atoms. The second strongest antiferromagnetic interaction occurs between the copper(II) ions bridged by two chlorido ligands, expressed as *J*_2_ (=−38.5 cm^−1^). The third interaction, considered to be ferromagnetic, was between the copper(II) ions bridged by one chlorido ligand, expressed as *J*_3_ (=15.3 cm^−1^). This order, |*J*_1_| > |*J*_2_| > |*J*_3_|, is consistent with the order of Cu···Cu distances. That is, the shorter the distance, the stronger the interaction, although this is a rough estimation. From the viewpoint of the molecular orbital theory, the magnetic orbitals should be on the basal planes of square–pyramidal coordination geometries around copper(II) ions. Using the local coordinates, each magnetic orbital is expressed as *d_x_*^2^_–*y*_^2^, assuming the local *z*-axis to the apical direction and the local *x*- and *y*-axes to the donor atom directions in the basal plane. Since the two local magnetic orbitals of the pair of copper(II) ions in the same dinucleating ligand are almost in the same plane, the strongest antiferromagnetic interaction is expected between Cu_A1_ and Cu_B1_ in Figure 4b, expressed as *J*_1_. In this way, the obtained interaction parameters, *J*_1_, *J*_2_, and *J*_3_, can be reasonably understood. Other obtained parameters are also reasonable for copper(II) complexes.

### 2.4. Density Functional Theory (DFT) Calculation

**Figure 5:**
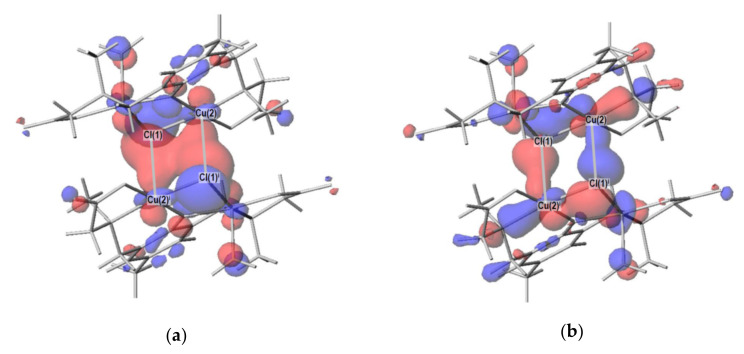

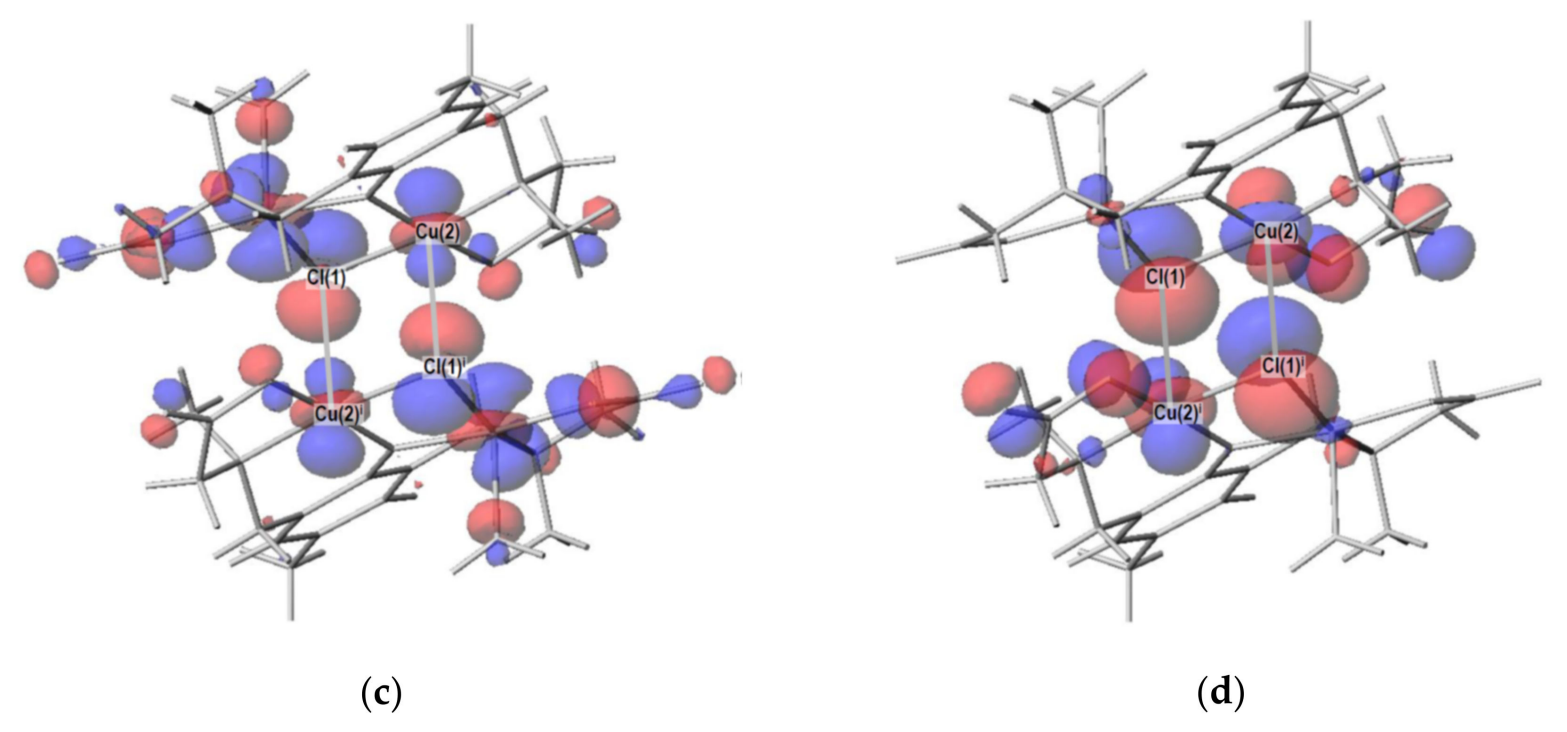
Molecular orbitals in **1′** with respect to the Cu(2)–Cl(1)^i^ coordination bond: (**a**) *gerade* bonding orbital; (**b**) *ungerade* bonding orbital; (**c**) *gerade* anti-bonding orbital; (**d**) *ungerade* anti-bonding orbital.

In order to confirm the bonding nature around copper(II) ions in the complexes, density functional theory (DFT) calculations were conducted. In particular, the Cu(2)–Cl(1)^i^ distance (2.8011(13) Å) in **1′** seems to be long, and whether this bond is a coordination bond or an ionic bond should be clarified based on the molecular orbital theory. As a result of the DFT calculation, bonding and anti-bonding orbitals were observed for the Cu(2)–Cl(1)^i^ bond (Figure 5), indicating the covalent nature of the bond. Note that the coordination bond is the same as the covalent bond from the viewpoint of molecular orbital theory [13], although they were long considered to be different. In **1′**, the Cu(2)–Cl(1)^i^ bond is formed using the *d_z_*^2^ atomic orbital of Cu(2) atom and *p_z_* atomic orbital of Cl(1)^i^, where the *z* direction is defined as the local apical direction around the Cu(2) atom. In this way, the five typical coordination bonds around each copper(II) ion were confirmed by the DFT calculations for both complexes **1′** and **2**. In addition, molecular orbitals with bonding and anti-bonding characters were found for each of the three weak coordination bonds (Cu(1)–O(4)^i^ and Cu(2)–O(3)^ii^ in **1′** and Cu(1)–O(5)^i^ in **2**) discussed in Section 2.2, although the overlap of atomic orbitals involved was smaller than that of typical coordination bonds.

Another purpose of the DFT calculation is to confirm the magnetic orbitals possessing the unpaired electrons. The doubly degenerate highest occupied molecular orbitals and doubly degenerate lowest unoccupied molecular orbitals were all found to be based on the local *d_x_*^2^_–*y*_^2^ atomic orbitals of copper(II) ions orientated along the four donor atoms in the basal plane of each copper(II) coordination polyhedron. In addition, the strong antiferromagnetic interaction (*J*_1_) between Cu(1) and Cu(2) was understood by the phenolato and chlorido bridges, considering the overlaps of the local magnetic orbitals and bridging atomic orbitals.

### 2.5. Electronic Spectra and Structure in Aqueous Solution

**Figure 6:**
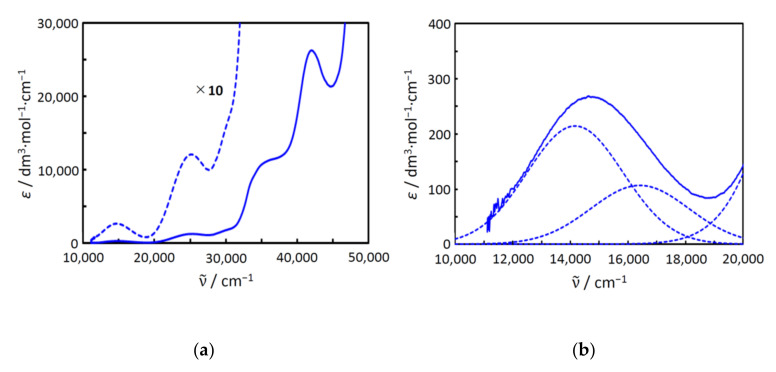
Electronic spectra of **1′** in water: (**a**) spectra in the range of 10,000–50,000 cm^−1^; (**b**) the first band with the Gaussian spectral components.

**Figure 7:**
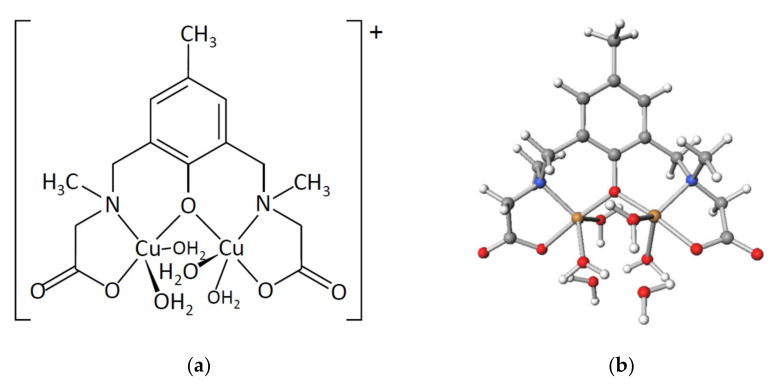
Proposed dinuclear copper(II) structure in aqueous solution: (**a**) chemical structure of [Cu_2_(*sym*-cmp)(H_2_O)_4_]^+^; (**b**) DFT-based optimized structure of {[Cu_2_(*sym*-cmp)(H_2_O)_4_]·2H_2_O}^+^.

The electronic spectra of complex **1** were measured in water (Figure 6). Judging from the molar conductance in water (see Section 3.2), the chlorido ligands were thought to be dissociated in water to break the tetranuclear copper(II) structure. However, judging from the green color of the solution, copper(II) ions were thought to be remain in the dinucleating ligand (*sym*-cmp)^3−^. The spectra are shown in Figure 6. The first band at around 15,000 cm^−1^ can be assigned to the *d*–*d* band. As a result of the Gaussian curve fitting, the first band was found to consist of two absorption components at around 14,200 cm^−1^ and 16,400 cm^−1^. The intensity of the first component is slightly larger than that of the second one, and this pattern is typical of trigonal–bipyramidal copper(II) complexes. Under the *D*_3*h*_ symmetry, the first and the second components are assigned to ^2^*A*_1′_ → ^2^*E*’ and ^2^*A*_1′_ → ^2^*E*”, respectively. The electronic spectra of the complexes containing trigonal–bipyramidal [CuCl_5_]^3−^ anions were investigated earlier [14], and the positions of the components of **1** are found to be reasonable, considering the ligand-field strengths. This spectral feature of **1** suggests that all the copper(II) ions have almost the same structures, incorporated in the dinucleating ligand to form [Cu_2_(*sym*-cmp)(H_2_O)_4_]^+^ species in aqueous solutions (Figure 7a). Generally, additional chlorido bridge or hydroxido bridge is expected to be formed between copper(II) ions; however, in this case, such an anionic bridging ligand is not so favorable because of the negative charge on the carboxylate side chains of the (*sym*-cmp)^3−^ ligand. This idea is consistent with the facile dissociation of the chlorido ligands in aqueous solutions confirmed by the conductivity measurement described previously.

The proposed species in an aqueous solution, [Cu_2_(*sym*-cmp)(H_2_O)_4_]^+^, was confirmed on the basis of the DFT calculation assuming the water environment. Judging from the electronic spectra, the coordination geometry was trigonal–bipyramidal. When the [Cu_2_(*sym*-cmp)(H_2_O)_4_]^+^ was structurally optimized with two additional water molecules, the proposed trigonal–bipyramidal structure was successfully reproduced as shown in Figure 7b. The distortion parameter *τ* around the copper(II) ions fell in the range of 85.4–86.0%, which is considered to be trigonal–bipyramidal. This DFT result is strong evidence of the proposed structure. The proposed structure, including two copper(II) ions with four water molecules, is in concordance with the previously proposed structures for dinuclear cobalt(II) and nickel(II) complexes with a related dinucleating ligand in aqueous solutions [7].

### 2.6. Structures of Metal Complexes and Ligand Design

Similar to the popular (5-Me-hxta)^5−^ ligand, possessing four carboxylate chelating side chains, the (*sym*-cmp)^3−^ ligand in this study possesses two carboxylate chelating side chains. Although the number of side chains is reduced, the ligand is still capable of holding two copper(II) ions in an aqueous solution. With the reduction of the ligand-occupying sites, a larger substrate is expected to be incorporated at the coordination sites. On the other hand, changes in the ligand charge can lead to a variety of metal complex structures. For example, 2,6-bis[(2-hydroxyethyl)methylaminomethyl]-4-methylphenolate ((*sym*-hmp)^−^) [15] and 4-chloro-2,6-bis[(2-hydroxyethyl)methylaminomethyl] phenolate ((*sym*-hcp)^−^) [16] each have a skeletal structure very similar to (*sym*-cmp)^3−^ ligand; however, the obtained complex structures are different. The (*sym*-hmp)^−^ and (*sym*-hcp)^−^ ligands form 2:2 (ligand:metal) metal complexes, [M_2_(*sym*-hmp)_2_] (M = Mg(II) [17], Mn(II) [16], Co(II) [15,18,19], Ni(II) [20,21], Zn(II) [21]), while the (*sym*-cmp)^3−^ ligand forms 2:4 (ligand:metal) metal complexes, [M_4_(*sym*-hmp)_2_X_2_Y_2_] (M = Cu(II); X = Cl^−^, CH_3_O^−^; Y = H_2_O, CH_3_OH), as presented in this paper. This difference is thought to be caused by the difference in ligand charge. Concludingly, the skeletal structure, charge, bulkiness, etc., of the ligand can give rise to various metal complex structures. The knowledge of creating various controlled structures will also enable the development of metal-organic frameworks (MOFs) and is expected to be useful in various applications [22,23,24,25,26,27,28,29], beyond the use of molecular complexes as homogeneous catalysts.

## 3. Materials and Methods

### 3.1. Measurements

Elemental analyses (C, H, and N) were performed at the Elemental Analysis Service Centre of Kyushu University. Copper(II) ions were quantified by titration with ethylenediaminetetraacetic acid in the presence of hydrochloric acid, using murexide as an indicator. IR spectra were recorded on a Jasco FT/IR-4100 FT-IR spectrometer. ^1^H and ^13^C NMR spectra (400 MHz) on a Bruker-Biospin AV 400 NMR spectrometer in D_2_O, electrospray ionization (ESI) mass spectra on a Waters Quattro micro API mass spectrometer in methanol, and electronic spectra on Jasco V-560 (200–800 nm) and Hitachi 330 (800–2000 nm) spectrophotometers. Molar conductance was measured in H_2_O on a DKK AOL-10 conductivity meter at room temperature. Magnetic susceptibility measurements were performed with a Quantum Design MPMS-7 SQUID magnetometer in the temperature range from 1.9 to 300 K with a static field of 5 kOe. The polycrystalline samples were ground into fine powders in an agate mortar. The sample was wrapped with aluminum foil. Data were corrected for paramagnetism of the aluminum foil. The susceptibilities were corrected for the diamagnetism of the samples by means of Pascal’s constants.

### 3.2. Materials

All the chemicals were commercial products and were used as supplied. Methanol, ethanol, copper(II) nitrate–water (1/3), copper(II) chloride–water (1/2), paraformaldehyde, *p*-cresol, sodium hydroxide, lithium hydroxide–water (1/1), phosphorus pentoxide, 2-propanol, ethylenediaminetetraacetic acid, and hydrochloric acid were supplied by Nacalai Tesque Inc. (Kyoto, Japan). Sarcosine and murexide were supplied by Tokyo Chemical Industry Co., Ltd. (Tokyo, Japan).

### 3.3. Preparations

Disodium 2,6-bis{[*N*-(carboxylatomethyl)-*N*-methyl-amino]methyl}-4-methylphenol—water (1/3) (Na_2_H(*sym*-cmp)·3H_2_O). To an aqueous solution (20 mL) containing *p*-cresol (5.41 g, 50 mmol), NaOH (6.10 g, 153 mmol), sarcosine (8.95 g, 100 mmol), and paraformaldehyde (3.00 g, 100 mmol) were added ethanol (20 mL) and the resulting solution was refluxed for 1 week. Ethanol and water were removed by evaporation to give Na_2_H(*sym*-cmp) as a colorless powder. Yield 14.25 g (70%). (Found: C, 44.00; H, 6.45; N, 7.00; Calc. for C_15_H_20_N_2_Na_2_O_5_·3H_2_O: C, 44.10; H, 6.40; N, 6.85). Selected IR data [*ṽ*/cm^−1^] using KBr disk (Figure S5): 3325, 2985, 2955, 2830, 1585, 1420, 1405, 1365, 1330, 1245, 855, 770, 715, 665. ^1^H NMR in D_2_O: *δ* 2.13 (s, 3 H), 2.27 (s, 6 H), 3.13 (s, 4 H), 3.67 (s, 4 H), 6.94 (s, 2 H). ^13^C NMR in D_2_O: *δ* 19.29, 40.97, 56.74, 59.86, 122.77, 127.10, 130.81, 155.36, 177.46. ESI mass spectrum in MeOH: *m*/*z* 309, [H_2_(*sym*-cmp)]^−^; 331, [NaH(*sym*-cmp)]^−^. Molar conductance in H_2_O [*Λ*/S·cm^2^·mol^−1^] 19.

[Cu_4_(*sym*-cmp)_2_Cl_2_(H_2_O)_2_]·2H_2_O **1**. To a methanolic solution (5 mL) of copper(II) chloride—water (1/2) (0.34 g, 2.0 mmol) was added a methanolic solution (5 mL) of Na_2_H(*sym*-cmp)·3H_2_O (0.38 g, 0.93 mmol), and the resulting solution was stirred for 30 min to give the precipitation of green powder. Recrystallized from methanol, washed with methanol, and dried in vacuo over P_2_O_5_. Yield 0.30 g (64%) (Found: C, 35.20; H, 4.60; N, 5.50; Cu, 24.70; Calc. for C_30_H_42_Cl_2_Cu_4_N_4_O_12_·2H_2_O: C, 35.60; H, 4.60; N, 5.55; Cu, 25.10). Selected IR data [*ṽ*/cm^−1^] using KBr disk (Figure S6): 3050-3700, 3010, 2970, 2920, 2865, 2815, 1630, 1475, 1385, 1195, 870, 545, 465. Molar conductance in H_2_O [*Λ*/S·cm^2^·mol^−1^] 250 (1.1 × 10^−3^ mol·dm^−3^), 250 (5.7 × 10^−4^ mol·dm^−3^), 290 (1.1 × 10^−4^ mol·dm^−3^).

[Cu_4_(*sym*-cmp)_2_(CH_3_O)_2_(CH_3_OH)_2_]·2C_3_H_7_OH·2CH_3_OH **2**. To a methanolic solution (5 mL) of copper(II) nitrade—water (1/3) (0.24 g, 0.99 mmol) was added a methanolic solution (5 mL) of Na_2_H(*sym*-cmp)·3H_2_O (0.19 g, 0.47 mmol), and the resulting solution was refluxed for 2 h to give the precipitation of white powder. After filtration, the addition of 2-propanol (5 mL) resulted in the precipitation of dark-green powder. Recrystallized from methanol/2-propanol to give dark-green crystals. Yield 0.09 g (31%).

### 3.4. Crystallography

Crystallographic data are summarized in Table 5. Single crystals of **1′** suitable for X-ray analysis were obtained from a methanolic solution of **1**. Single crystals of **2** were obtained by slow diffusion of 2-propanol to a methanolic solution of the crude product. Single-crystal X-ray diffraction data were obtained with a Rigaku XtaLAB AFC11 diffractometer with graphite-monochromated Mo Kα radiation (λ = 0.71073 Å). A single crystal was mounted with a glass capillary and flash-cooled with a cold N2 gas stream. Data were processed using the CrysAlisPro software packages. The structure was solved by intrinsic phasing methods using the SHELXT [30] software packages and refined on F2 (with all independent reflections) using the SHELXL [31] software packages. The non-hydrogen atoms were refined anisotropically, and hydrogen atoms were refined using the riding model. Complex 2 was refined as a two-component twin with only the non-overlapping reflections of component 1 and was refined using the hklf 5 routine with all reflections of component 1 (including the overlapping ones). The Cambridge Crystallographic Data Centre (CCDC) deposition numbers are included in Table 5.

### 3.5. Computation

Magnetic analyses and magnetic simulation were conducted using the MagSaki(TetraW9.2.0Cu) programs of the MagSaki series. DFT computations were performed using the GAMESS program [32,33] on Fujitsu PRIMERGY CX2550/CX2560 M4 (ITO super computer system) at Kyushu University. Calculations were performed with LC-BOP/6-31G [34]. When considering the solvent effect, the polarizable continuum model (PCM) method was used.

## 4. Conclusions

A water-soluble dinucleating ligand, (*sym*-cmp)^3^^−^, was prepared, and two dimer-of-dimers type tetranuclear copper(II) complexes with (*sym*-cmp)^3^^−^ were prepared. The structures of the complexes were crystallographically characterized, and [Cu_4_(*sym*-cmp)_2_Cl_2_(H_2_O)_2_] and [Cu_4_(*sym*-cmp)_2_(CH_3_O)_2_(CH_3_OH)_2_] complexes were found to have the defect cubane tetranuclear copper(II) core structures. In the complexes, each copper(II) ion has a five-coordinate square-pyramidal coordination geometry, and the coordination bonds were confirmed by the DFT calculation, whereby we found the bonding and anti-bonding molecular orbitals. The cryomagnetic measurement was conducted to find the overall antiferromagnetic interaction in the tetranuclear copper(II) structure. The observed magnetic data were successfully simulated with the tetranuclear model to find reasonable magnetic parameters. Judging from the molar conductance and the electronic spectra, the tetranuclear structure was found to be broken in an aqueous solution, but the dinuclear copper(II) structure, [Cu_2_(*sym*-cmp)(H_2_O)_4_]^+^, was considered to be maintained in an aqueous solution. This proposed structure was supported by DFT calculation.

## Figures and Tables

**Table 1:** Selected distances for **1′**.

Atom–Atom ^1^	Distance/Å	Atom–Atom ^1^	Distance/Å
Cu(1)–Cl(1)	2.3638(14)	Cu(1)–O(1)	1.937(3)
Cu(1)–O(2)	1.926(3)	Cu(1)–O(6)	2.214(4)
Cu(1)–N(1)	1.998(4)	Cu(1)–O(4)^i^	3.208(4)
Cu(2)–Cl(1)	2.3113(14)	Cu(2)–O(1)	1.942(3)
Cu(2)–O(4)	1.936(3)	Cu(2)–N(2)	2.003(4)
Cu(2)–Cl(1)^i^	2.8012(14)	Cu(2)–O(3)^ii^	2.804(4)
Cu(1)···Cu(2)	3.1274(8)	Cu(1)···Cu(1)^i^	5.8824(12)
Cu(1)···Cu(2)^i^	3.8024(9)	Cu(2)···Cu(2)^i^	3.7248(13)

^1^ Symmetry code: ^i^ (−*x* + 1/2, −*y* + 1/2, −*z* + 1), ^ii^ (−*x* + 1/2, *y* + 1/2, −*z* + 3/2).

**Table 2:** Selected angles for **1′**.

Atom–Atom–Atom ^1^	Angle/°	Atom–Atom–Atom ^1^	Angle/°
Cl(1)–Cu(1)–O(1)	83.38(11)	Cl(1)–Cu(1)–O(2)	94.87(10)
Cl(1)–Cu(1)–O(6)	94.72(10)	Cl(1)–Cu(1)–N(1)	161.10(12)
Cl(1)–Cu(1)–O(4)^i^	77.29(7)	O(1)–Cu(1)–O(2)	173.59(15)
O(1)–Cu(1)–O(6)	90.83(14)	O(1)–Cu(1)–N(1)	94.06(15)
O(1)–Cu(1)–O(4)^i^	92.89(12)	O(2)–Cu(1)–O(6)	95.46(14)
O(2)–Cu(1)–N(1)	85.61(15)	O(2)–Cu(1)–O(4)^i^	80.71(12)
O(6)–Cu(1)–N(1)	104.06(15)	O(6)–Cu(1)–O(4)^i^	170.73(12)
N(1)–Cu(1)–O(4)^i^	84.16(13)	Cl(1)–Cu(2)–O(1)	84.68(11)
Cl(1)–Cu(2)–O(4)	95.38(11)	Cl(1)–Cu(2)–N(2)	172.22(13)
Cl(1)–Cu(2)–Cl(1)^i^	86.96(5)	Cl(1)–Cu(2)–O(3)^ii^	84.35(8)
O(1)–Cu(2)–O(4)	175.93(15)	O(1)–Cu(2)–N(2)	94.72(16)
O(1)–Cu(2)–Cl(1)^i^	89.05(11)	O(1)^i^–Cu(2)–O(3)^ii^	130.04(9)
O(4)–Cu(2)–N(2)	84.66(15)	O(4)–Cu(2)–Cl(1)^i^	95.02(11)
O(4)–Cu(2)–O(3)^ii^	88.47(12)	N(2)–Cu(2)–Cl(1)^i^	100.79(12)
N(2)–Cu(2)–O(3)^ii^	87.87(14)	Cl(1)^i^–Cu(2)–O(3)^ii^	170.91(8)
Cu(1)–Cl(1)–Cu(2)	83.96(5)	Cu(1)–O(1)–Cu(2)	107.46(17)
Cu(1)–Cl(1)–Cu(2)^i^	94.44(4)	Cu(2)–Cl(1)–Cu(2)^i^	93.04(5)

^1^ Symmetry code: ^i^ (−*x* + 1/2, −*y* + 1/2, −*z* + 1), ^ii^ (−*x* + 1/2, *y* + 1/2, −*z* + 3/2).

**Table 3:** Selected distances for **2**.

Atom–Atom ^1^	Distance/Å	Atom–Atom ^1^	Distance/Å
Cu(1)–O(1)	1.930(3)	Cu(1)–O(2)	1.933(3)
Cu(1)–O(3)	1.981(3)	Cu(1)–O(7)	2.331(3)
Cu(1)–N(1)	2.008(4)	Cu(1)–O(5)^i^	2.856(3)
Cu(2)–O(1)	1.930(3)	Cu(2)–O(3)	1.999(3)
Cu(2)–O(5)	1.936(3)	Cu(2)–N(2)	2.019(4)
Cu(2)–O(3)^i^	2.305(3)	Cu(2)···O(6)^ii^	3.3478(3)
Cu(1)···Cu(2)	3.0138(8)	Cu(1)···Cu(1)^i^	5.5123(12)
Cu(1)···Cu(2)^i^	3.3743(9)	Cu(2)···Cu(2)^i^	3.2483(11)

^1^ Symmetry code: ^i^ (−*x* + 1, −*y* + 1, −*z* + 1), ^ii^ (−*x* + 2, −*y* + 1, −*z* + 1).

**Table 4:** Selected angles for **2**.

Atom–Atom–Atom ^1^	Angle/°	Atom–Atom–Atom ^1^	Angle/°
O(1)–Cu(1)–O(2)	174.14(13)	O(1)–Cu(1)–O(3)	79.22(13)
O(1)–Cu(1)–O(7)	88.52(13)	O(1)–Cu(1)–N(1)	93.40(14)
O(1)–Cu(1)–O(5)^i^	89.28(12)	O(2)–Cu(1)–O(3)	100.38(14)
O(2)–Cu(1)–O(7)	97.33(13)	O(2)–Cu(1)–N(1)	85.53(14)
O(2)–Cu(1)–O(5)^i^	84.96(11)	O(3)–Cu(1)–O(7)	90.78(13)
O(3)–Cu(1)–N(1)	164.26(14)	O(3)–Cu(1)–O(5)^i^	76.54(11)
O(7)–Cu(1)–N(1)	103.00(14)	O(7)–Cu(1)–O(5)^i^	167.32(11)
N(1)–Cu(1)–O(5)^i^	89.59(13)	O(1)–Cu(2)–O(3)	78.76(13)
O(1)–Cu(2)–O(5)	170.63(13)	O(1)–Cu(2)–N(2)	93.49(14)
O(1)–Cu(2)–O(3)^i^	96.27(13)	O(3)–Cu(2)–O(5)	99.19(13)
O(3)–Cu(2)–N(2)	164.05(15)	O(3)–Cu(2)–O(3)^i^	82.25(13)
O(5)–Cu(2)–N(2)	86.18(14)	O(5)–Cu(2)–O(3)^i^	92.48(12)
N(2)–Cu(2)–O(3)^i^	112.69(13)	Cu(1)–O(1)–Cu(2)	102.66(15)
Cu(1)–O(3)–Cu(2)	98.45(14)	Cu(1)–O(3)–Cu(2)^i^	103.63(13)
Cu(2)–O(3)–Cu(2)^i^	97.75(12)		

^1^ Symmetry code: ^i^ (−*x* + 1, −*y* + 1, −*z* + 1).

**Table 5:** Crystallographic data and refinement parameters of **1′** and **2**.

Complex	1′	2
Empirical formula ^1^	C_16.2_H_27.6_ClCu_2_N_2_O_8.1_	C_21_H_38_Cu_2_N_2_O_9_
Formula weight ^1^	542.53	589.61
Crystal system	Monoclinic	triclinic
Space group	*C*2/*c*	$P\bar{1}$
*a*/Å	27.0693(14)	8.5827(6)
*b*/Å	13.2690(5)	13.0679(8)
*c*/Å	13.1356(7)	13.2862(7)
α/°	90	115.021(6)
β/°	100.422(5)	102.208(5)
γ/°	90	115.021(6)
*V*/Å ^3^	4640.2(4)	1263.54(15)
*Z* ^1^	8	2
Crystal dimensions/mm	0.070 × 0.050 × 0.030	0.130 × 0.057 × 0.038
*T*/K	100	100
λ/Å	0.71073	0.71073
*ρ*_calcd_/g·cm^−3^	1.553	1.645
*µ*/mm^−1^	1.990	1.734
*F*(000)	2229	616
2*θ*_max_/◦	55	55
No. of reflections measured	9520	16014
No. of independent reflections	9520 (Rint = 0.0623)	5771 (Rint = 0.0770)
Data/restraints/parameters	9520/3/296	5771/45/341
*R*1 ^2^ [*I* > 2.00 σ(*I*)]	0.0575	0.0674
*wR*2 ^3^ (all reflections)	0.1664	0.1635
Goodness of fit indicator	1.020	0.992
Highest peak, deepest hole/e Å^−3^	1.787, −0.647	1.669, −1.068
CCDC deposition number	2130618	2130619

^1^ Based on dinuclear unit, ^2^ *R*1 = Σ||Fo| − |Fc||/Σ|Fo|, ^3^ *wR*2 = [Σ(*w*(Fo^2^ − Fc^2^)^2^)/Σ*w*(Fo^2^)^2^]^1/2^.

## Data Availability

The crystallographic data are available from the Cambridge Crystallographic Data Centre (CCDC). Other data not presented in Supplementary Materials are available on request from the corresponding author.

## Supplementary Materials

The following supporting information can be downloaded. Figure S1: ^1^H NMR of Na_2_H(sym-cmp)·3H_2_O., Figure S2: ^13^C NMR of Na_2_H(sym-cmp)·3H_2_O, Figure S3: ESI-mass spectra of Na_2_H(sym-cmp)·3H_2_O, Figure S4: Isotope pattern for Na_2_H(sym-cmp)·3H_2_O: (a) observed for m/z 309; (b) theoretical for m/z 309; (c) observed for m/z 331; (d) theoretical for m/z 331., Figure S5: IR spectra of of Na_2_H(sym-cmp)·3H_2_O, Figure S6: IR spectra of 1.
