# Supplementary material for: Structures of Dimer-of-Dimers Type Defect Cubane Tetranuclear Copper(II) Complexes with Novel Dinucleating Ligands
Source: Molecules. 2022 Jan 17;27(2):576. doi: 10.3390/molecules27020576 (PMC8780986; doi:10.3390/molecules27020576)
Supplement: Supplementary file 1 [file molecules-27-00576-s001.zip › molecules-1548432-supplementary.pdf]

Supplementary Materials

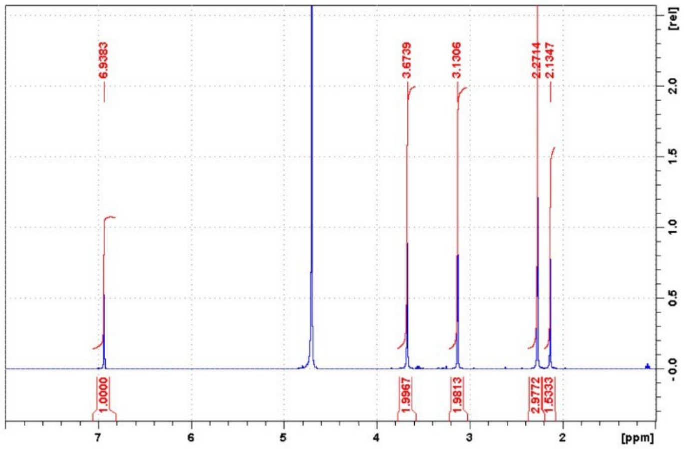

Figure S1. ^{1}H NMR of Na_2H(*sym*-cmp)·$3H_2O$.

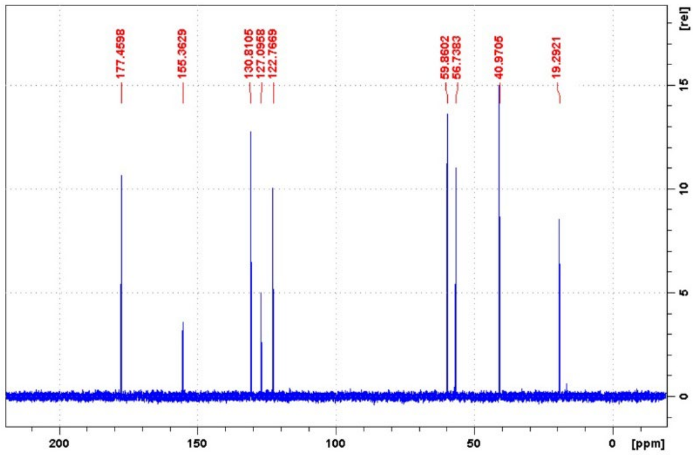

Figure S2. ^{13}C NMR of Na_2H(*sym*-cmp)·$3H_2O$.

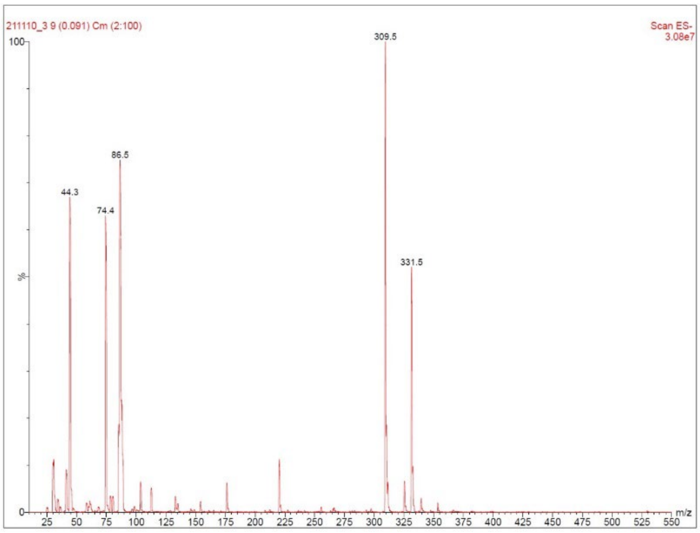

Figure S3. ESI-mass spectra of Na_2H(*sym*-cmp)·$3H_2O$.

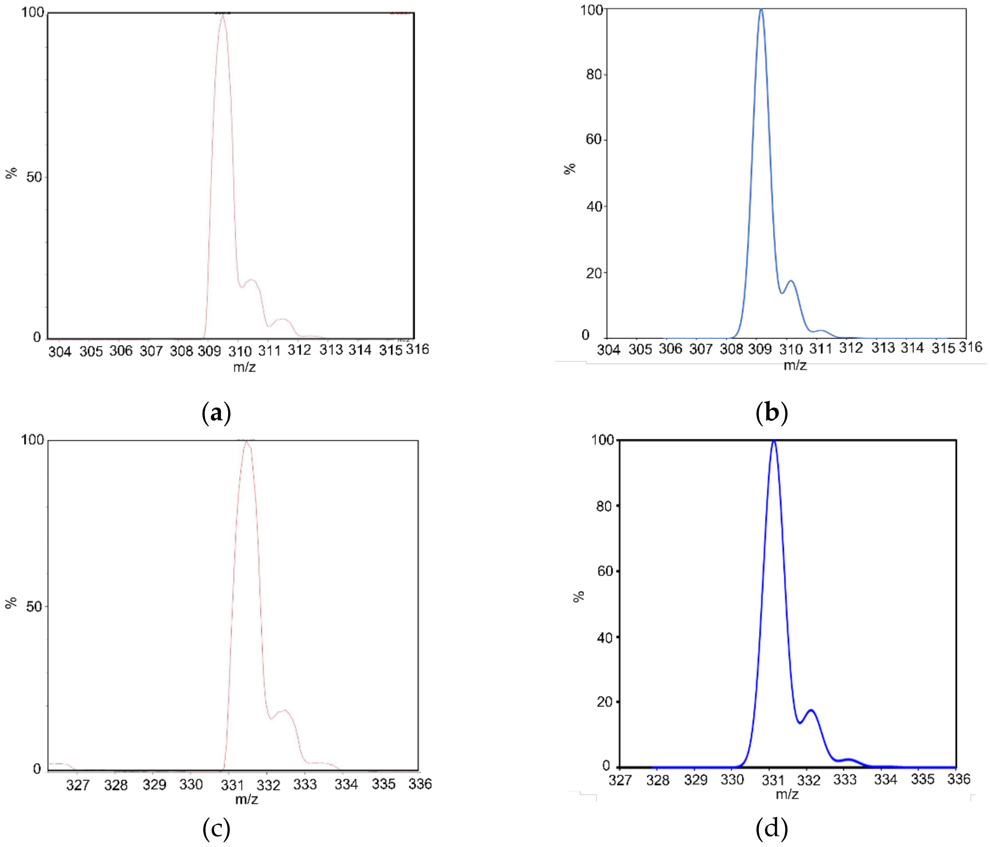

Figure S4. Isotope pattern for Na_2H(*sym*-cmp)·$3H_2O$: (a) observed for *m*/*z* 309; (b) theoretical for *m*/*z* 309; (c) observed for *m*/*z* 331; (d) theoretical for *m*/*z* 331.

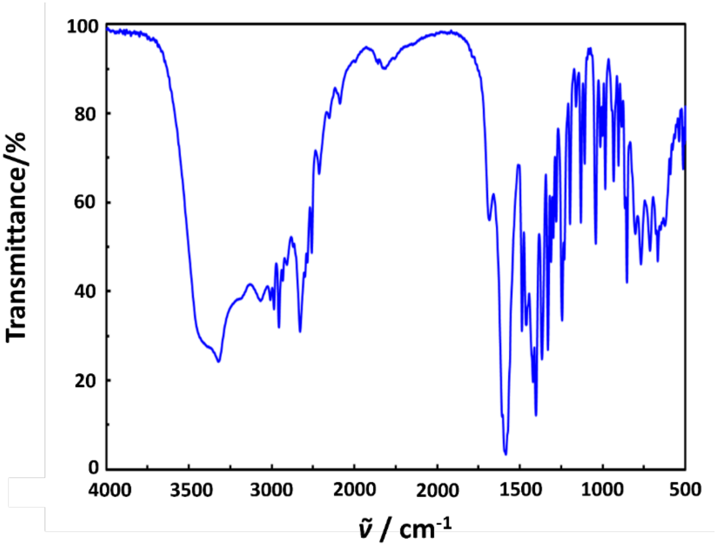

Figure S5. IR spectra of of Na_2H(*sym*-cmp)·$3H_2O$.

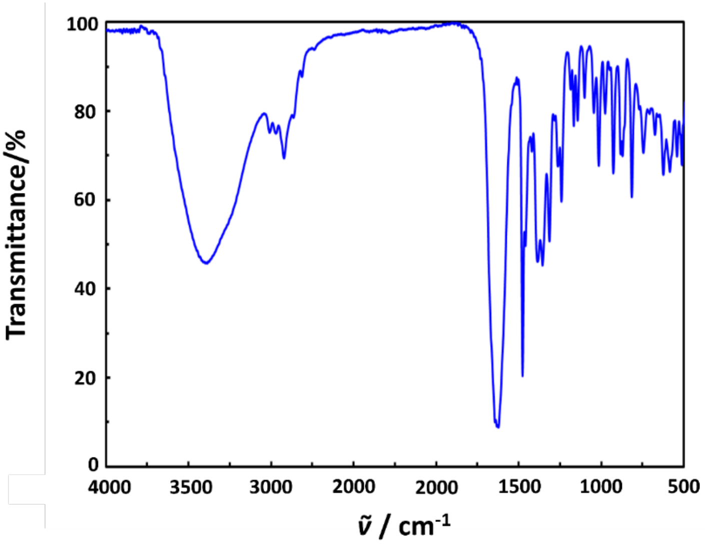

Figure S6. IR spectra of **1**.
